# Evaluation of Self-Help Cognitive Behavioural Therapy for Children’s Dental Anxiety in General Dental Practice

**DOI:** 10.3390/dj7020036

**Published:** 2019-04-01

**Authors:** Sarah Bux, Jenny Porritt, Zoe Marshman

**Affiliations:** 1 Clapton Dental Surgery, London NW3 6BP, UK; 2 Department of Psychology, Sociology and Politics, Sheffield Hallam University, Sheffield S1 1WB, UK; j.porritt@shu.ac.uk; 3 School of Clinical Dentistry, University of Sheffield, Sheffield S10 2TA, UK; Z.Marshman@sheffield.ac.uk

**Keywords:** dental anxiety, paediatric dentistry, cognitive behaviour therapy, behaviour management, primary care, service evaluation

## Abstract

Dental anxiety is very common; however, there is a lack of studies focusing on reducing children’s dental anxiety. One such initiative, the guided self-help cognitive behavioural therapy (CBT) resources ‘Your teeth, you are in control’, reduces dental anxiety in children attending paediatric dentistry clinics. This service evaluation aims to investigate whether such CBT resources reduce children’s dental anxiety when implemented in general dental practice. A convenience sample of children was given the resources by their dental practitioner. There was no control group. Children completed the Children’s Experiences of Dental Anxiety Measure (CEDAM) prior to using the resources and on completion of a course of dental treatment. Overall, 84 children were involved, with a mean age of 10.9 years; 48 were female and 59 were living in the most deprived area of England. At baseline the mean CEDAM score was 20.3, and on receiving the resource and completing treatment the mean CEDAM score was 16.4, showing a significant reduction in dental anxiety (t = 14.6, (*df* = 83), *p* < 0.001, 95% CI: 3.4–4.4). The items that improved the most were worry over having dental treatment and dental treatment being painful. The service evaluation indicates a reduction in child dental anxiety following the use of CBT resources in general practice. Further evaluation, preferably a randomised controlled trial, is needed.

## 1. Introduction

Dental fear and anxiety vary across a continuum, from very mild to severe fear, with around 10% of children and young people experiencing severe dental anxiety [1]. Children with higher levels of dental anxiety tend to have an increased prevalence of untreated decay [2] and worse quality of life than less dentally anxious children [2,3]. One possible reason for this is that children who are fearful of dental treatment are more likely to avoid or delay dental care due to their fear and therefore this group of patients will typically experience more oral pain and functional problems [4]. Children report using a variety of strategies to persuade parents/carers into cancelling dental appointments, which include claiming to be unwell and downplaying their dental problems [5]. The study by Luoto et al. revealed that it is specifically the fear of dental treatment procedures which are related to worse oral health-related quality of life in children [3]. Dental anxiety and avoidance of dental treatment are factors associated with worse oral health outcomes in children as young as 5 years old [6]. 

In the UK, the vast majority of dental care for children is provided by general dental practitioners working in the National Health Service (NHS). However, treating children with dental anxiety within general dental practice can be time-consuming, stressful, and not well remunerated [7]. In addition, some dental team members are reluctant to carry out dental treatment on anxious patients for fear of negatively reinforcing their dental anxiety. Consequently, children with dental anxiety where usual behaviour management techniques have proved unsuccessful are often referred to specialist dental services for pharmacological interventions, for example, conscious sedation or general anaesthesia. While these pharmacological approaches enable the required dental treatment to be carried out, they do not address the children’s dental anxiety, which often continues into adulthood [8].

More recently, it has been recognised that greater effort should be directed towards psychological interventions to reduce patients’ dental anxiety in the long term [9]. A recent systematic review indicated that cognitive behavioural therapy (CBT) (e.g., positive self-talk, distraction, relaxation training, and exposure response prevention) resulted in lower levels of dental anxiety and better cooperation compared to various other pharmacological and behavioural management techniques (e.g., sedation, general anaesthesia, modelling, and restraint) [10]. Half of the studies included in the review delivered only one session of CBT immediately prior to dental treatment. These findings are promising and provide preliminary evidence for the effectiveness of low-level CBT interventions for the management of children’s dental anxiety. It is therefore important to consider how CBT-based anxiety management techniques can be integrated into general dental practice in a way which is feasible and acceptable to both patients and dental teams, whose practice is subject to significant time and resource constraints. Guided self-help CBT interventions involve practitioners supporting their patients to work through self-help resources which enable them to learn skills that will allow them to better manage their anxiety. Guided self-help interventions can be integrated into primary medical care as part of a ‘stepped care’ approach to anxiety management [11] and guided self-help CBT programs have been found to offer a promising psychological treatment for children’s anxiety problems in primary medical care [12].

### ‘Your Teeth, You Are in Control’

Guided self-help CBT approaches are increasingly being used to reduce anxiety, including dental anxiety. Resources entitled ‘Your teeth, you are in control’ were developed by a team of children, parents, and experts in paediatric dentistry, clinical child psychology, health psychology, dental public health, and CBT. It uses a person-centred approach targeting children with mild to moderate dental anxiety who require a course of dental treatment but have no urgent dental treatment needs [13]. The CBT components of the resources include: (1) challenging unhelpful thoughts by normalising dental anxiety and providing information, (2) enhancing control via a message to dentist proforma, the stop signal contract, and developing positive coping strategies, and (3) encouraging children to reflect on what went well and plan a small reward. The guided component of the guided self-help CBT approach is delivered by a dental professional.

Evaluations of these resources to date have found a significant reduction in dental anxiety and improvement in the quality of life when used with children attending community and hospital dental services [13]. One year later, 91% of these patients reported that the reduction in their dental anxiety was maintained, and described a change in their cognition, behaviours, and feelings that allowed them to better manage their anxiety [14]. However, these resources have yet to be evaluated in a general dental practice setting. 

This project aimed to conduct a service evaluation of the use of ‘Your teeth, you are in control’ among dentally anxious children attending a general dental practice in a deprived area of East London. The resources were introduced into the routine care pathway used in the dental practice in 2016, and as such there was no control group. This project was conducted to evaluate the impact of the resources on patient care. 

## 2. Materials and Methods

According to the UK ‘Policy Framework for Health and Social Care Research’ and the Health Research Authority decision tool, this project did not meet the definition of research and did not require ethical review by an NHS research ethics committee. Permission was received from the owner of the dental practice to conduct a service evaluation of the use of guided self-help CBT resources with children and their parents as part of the dental practice’s quality improvement programme. The project was conducted as a service evaluation; therefore, no control group or randomisation was used.

### 2.1. The Guided Self-Help CBT Resources

The general dental practitioner undertook online training on how to use the CBT resources (www.llttf.com/dental). The practitioner then started using the resource with children aged between 8 and 16 years who had self-reported dental anxiety on their initial examination appointment. Children were asked if they felt nervous or worried about coming to the dentist and whether they would feel nervous if they needed to have any dental treatment completed at subsequent appointments. The children who identified themselves as dentally anxious were then asked to complete the Children’s Experiences of Dental Anxiety measure (CEDAM) at the end of the first appointment to measure the extent of their anxiety. All of these children were offered the use of the CBT resource ‘Your teeth, you are in control’.

At the first appointment, children were given the paper-based self-help resources and parents were also given the accompanying resources on how to support their children with dental anxiety. The dentist provided the children and parents with an initial overview of the resources and explained that between appointments the resources could be used flexibly as the patient wished. The overview of the resources included the following:

- information for young people on the dental team and basic procedures;
- suggestions for coping tools that children can use such as a distraction (mind games, listening to their own music, squeezing a stress ball) and relaxation techniques;
- interactive activities such as a message to dentist proforma with a stop signal signed agreement, anxiety scores, and self-reflection on how things went. (A copy of the proforma can be found in the Supplementary Materials section).

**Figure 1:**
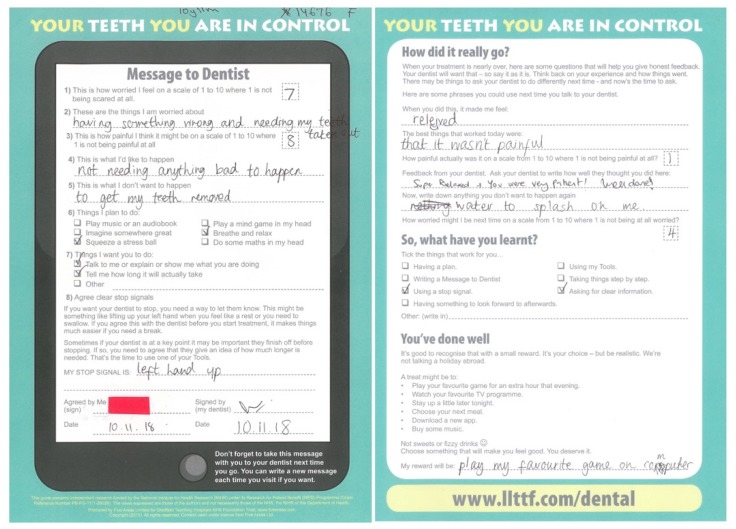
An image of a completed ‘Message to Dentist’ proforma, including the front and back pages.

At the start of each subsequent appointment, the dental practitioner reminded the patient of the information and coping tools available. The dentist discussed the relevant sections of the self-help resources and asked the child to complete the front page of the message to dentist proforma (Figure 1). Both the child and the dentist signed the stop signal agreement section of the proforma and shook hands. The dentist attempted to address points of concern and interest raised by the child on the proforma. In addition, all routine non-pharmacological behaviour management techniques were employed with patients during dental treatment (e.g., tell–show–do, voice control, behaviour shaping, and positive reinforcement).

The dental treatment delivered included preventive interventions (fluoride varnish and fissure sealants), restorations, and extractions. For patients where more than one procedure was required, the appointments for the simpler procedures were made first to allow for acclimatisation. 

On completion of dental treatment, the patient completed, with the dentist, the back page of the message to dentist proforma where both the child and dentist were asked to reflect and give feedback on how the session went. During the process, each child was asked, along with their parent/carer, how they would like to be rewarded for their progress. It was clarified that the appropriate rewards were inexpensive and simple, like watching their favourite TV show, choosing their next meal, or staying up later before bedtime. 

### 2.2. Data Collection

Children were asked to complete the Children’s Experiences of Dental Anxiety Measure (CEDAM) [15] before their dental treatment started and at the end of the course of the dental treatment. The CEDAM is based on a cognitive behavioural assessment model of anxiety and is a 14-item self-report measure which assesses the unhelpful thoughts, behaviours, physical symptoms, and feelings experienced by children who have higher levels of dental anxiety [15]. The measure was developed with children and aimed to provide a useful clinical assessment tool which can identify the internal components of dental anxiety experienced by children, as well as the factors that may be maintaining the child’s anxiety and should, therefore, be the target for change. Internal consistency (Cronbach’s alpha = 0.88) and excellent test–retest reliability (0.98) of the measure has been previously demonstrated [15]. Each item has the option of three possible responses specific to the question and is scored on a three-point ordinal scale (e.g., When I next visit the dentist I think… I will have a lot of control over what happens in the appointment (score = 1); I will have a bit of control over what happens in the appointment (score = 2); or I will not have any control over what happens in the appointment (score = 3)). Raw ordinal scores for each item are then totalled and converted into an interval score, allowing accurate calculation of change scores. The possible range of total scores is 14–42 (higher scores indicate a higher level of dental anxiety).

Other information gathered included the patient’s age at the time of appointment, their gender, and their postcode. The postcode was used to calculate Indices of Deprivation (2015) scores, which provide a set of relative measures of deprivation for small areas (Lower-Layer Super Output Areas) in England. Seven domains (e.g., income, employment, education/training, health, living environment, crime, and housing/services) were combined using a series of weights to produce the overall Index of Multiple Deprivation (IMD) score. Scores were converted into quintiles which rank areas from the most deprived to the least deprived and divide them into five equal groups. Lower scores reflect higher levels of deprivation.

### 2.3. Data Analysis

Raw CEDAM scores were converted into the interval scale as recommended by the developers of the measure [15]. Mean baseline and follow-up scores were then calculated and tests of difference were undertaken to establish whether there were any differences in children’s dental anxiety scores following the guided self-help CBT intervention and dental treatment. Change scores were calculated to investigate changes between assessment and follow-up scores for each item included in the questionnaire. Wilcoxon signed-rank tests were undertaken on this ordinal data to investigate whether these differences were significant.

## 3. Results

### 3.1. Sample

The guided self-help resources were provided to a total of 84 children; seven failed to return to the general dental practice for further dental treatment, and none refused to engage with the resources between November 2016 and November 2018. The majority of the patients were female (N = 48), and the mean age of patients was 10.9 years (SD = 2.0, range = 8.08–16.8 years). Seventy percent of the patients lived in the most deprived IMD quintile in England (N = 59), with the remaining patients living in the second most deprived IMD quintile in England (N = 19). Demographic data were missing for two patients and deprivation data were missing for an additional four patients. Patients attended an average of two dental treatment appointments between the baseline and the follow-up visits (range = 1–4).

### 3.2. Dental Anxiety

The mean baseline CEDAM interval score was 20.3 (SD = 2.2) and the mean follow-up CEDAM interval score was 16.4 (SD = 2.2). The internal consistency for the CEDAM in the current study was acceptable (baseline Cronbach’s alpha = 0.66). Following the children’s use of the guided self-help resource, 78 patients’ anxiety scores were decreased, five patients’ scores remained the same, and one patient’s anxiety score increased. Table 1 provides details of children’s CEDAM scores prior to and following the use of the guided self-help CBT resource and dental treatment. Wilcoxon signed-rank tests revealed that there were significant reductions in all of the CEDAM items between the baseline and the follow-up, with the exception of the item concerned with whether they would let the dentist look in their mouth (mean change score = 0 (SD = 0.3), three children’s scores decreased, Z = −0.56, *p* = 0.58). The items which decreased the most between the baseline and the follow-up included worry over the dentist telling them they need to have something done (mean change score = −0.6 (SD = 0.6), 44 children’s score decreased, Z = −5.98, *p* < 0.01), worry about the dental treatment being painful (mean change score = −0.6 (SD = 0.7), 43 children’s scores decreased, Z = −5.85, *p* < 0.01), and control over what will happen in the appointment (mean change score = −0.4 (SD = 0.7), 35 children’s scores decreased, Z = −5.05, *p* < 0.01). 

The data were normally distributed, and therefore a repeated *t*-test was undertaken to examine whether the difference between the baseline and the follow-up scores was statistically significant. The repeated *t*-test revealed that there was a significant reduction in the dental anxiety scores following the use of the guided self-help CBT resources within the dental practice (t = 14.6 (*df* = 83), *p* < 0.001, 95% CI: 3.4–4.4). The Cohen’s d effect size of the difference was large (Cohen’s d = 1.8). An ANCOVA revealed no significant interaction effects between the reduction in dental anxiety and the deprivation level of the children (F = 0.83 (*df* = 1), *p* = 0.37).

## 4. Discussion

Dental anxiety has a significant psychosocial impact on children and their families, with poor oral health outcomes carried into adulthood [4,16], compromised dental treatment decisions, and an increased reliance on costly specialist dental services. Guided self-help CBT provides an effective and child-centred approach to reducing child dental anxiety. However, previously this approach had only been evaluated in specialist paediatric dentistry settings [13]. This service evaluation found that the use of such resources reduced child dental anxiety when used in a general dental practice in a deprived area of London. 

The CEDAM was used to evaluate any changes in dental anxiety at the beginning and the end of the course of dental treatment, following children’s use of the guided self-help resource. The CEDAM is a self-report measure of children’s experience of dental anxiety which assesses the unhelpful thoughts, behaviours, physical symptoms, and feelings experienced by children. It was chosen for use in this service evaluation as it is the first measure developed which fully involved children in the development process [15]. However, with 14 questions a shorter version may be more appropriate for use as a routine clinical assessment measure of dental anxiety. The mean score reported in this general dental practice sample at the beginning of dental treatment (mean = 20.3) was slightly lower than the anxiety reported by children with dental anxiety who had been referred to specialist dental services (a paediatric dental unit and community dental service) for dental treatment (mean = 22.0) [15]. The items which had the largest decrease in score related to worries over having to have dental treatment and how painful dental treatment would be. These were also the items which decreased for most children. Improvements in these areas are promising given that the specific fear of pain and dental procedures is associated with dental avoidance and thus worse oral health and quality of life outcomes in children [3]. 

This guided self-help CBT resource, that proved useful in the general dental practice setting, provided age-appropriate information on specific procedures, involved the completion and discussion of the message to dentist proforma, the signing of the stop signal agreement, and encouraged children to use the distraction techniques. The stop signal agreement was popular as it allowed children to practice their own signature. While the use of stop signals is a commonly used behaviour management technique in paediatric dentistry [17], formalising the agreement has the added advantage of giving the child more choice and control. In terms of distraction, the children involved frequently took up the opportunity to choose which music they listened to and to use a stress ball. 

Guided self-help CBT approaches can provide an opportunity to develop patient–practitioner relationships because the activities often require the patient and dental professional to work together as part of a team. Effective communication and a patient’s trust in the dentist have been identified as key factors which can influence children’s dental anxiety [5]. Therefore, the ‘guided’ element of the self-help intervention used within the service may also have contributed to the reduction in children’s dental anxiety observed. There is flexibility in terms of the guided component of the self-help approach which allows different dental professionals to support their patients throughout the process in a way that works based on the setting, the severity of an individual patient’s dental anxiety, and the professional’s communication style. 

The previous evaluation of the use of the guided self-help resources identified the time to deliver the resources as a possible barrier to implementation. In this service evaluation, to save time, children were asked to complete the message to dentist proformas while waiting in reception and siblings were scheduled appointments together to allow the guidance to be delivered to several children at once. Future evaluations should consider investigating the impact of the guided self-help CBT resources on dental visiting behaviour as reducing avoidance of dental care has the potential to not only reduce unmet dental treatment need but also improve the efficiency of a dental practice. 

### Limitations of the Use of the Resources and the Service Evaluation

While the use of the resources was found to be beneficial, this approach may not be suitable for all children, particularly those who find reading difficult or who are unwilling to engage with the guided self-help CBT approach. In terms of readability, although the ability to read and enjoyment of reading do vary between children, the resources were developed and piloted [13] for use with children aged 8–16 years. One study found that approximately 30% of the children and their families refused to engage with or dropped out of the guided self-help CBT interventions for childhood anxiety within the primary medical care setting [12]. Therefore, it is important to consider how the needs of dentally anxious children who are not willing or able to engage with the self-help CBT approaches can be met within general dental practices. Over time, clinicians using the resources develop their own skills in rapidly identifying the patients who are most likely to engage from those who are less interested. Furthermore, for children with more severe dental anxiety, guided self-help CBT alone may not be sufficient but can be used as part of a care pathway which may also include pharmacological approaches. A variety of dental treatment variables (e.g., dental treatment type) and patient characteristics (e.g., clinical need, generalised anxiety) may influence how children respond to and benefit from the guided self-help approach. These potential confounders were not controlled for this service evaluation, but these factors should be considered within future evaluations of the guided self-help resources. 

The initial assessment of dental anxiety relied on children’s own self-reporting. This approach was similar to that used in the original development and evaluation of the resources [13] and as such no validated measures were used. Whilst the large effect size in the reduction of dental anxiety, following the use of the guided self-help resources, is comparable to effect sizes reported following the use of behavioural interventions [18], it is not possible to identify whether this reduction in anxiety was solely due to the use of the guided self-help resource or indeed whether this reduction was clinically meaningful. Currently, no measures of children’s dental anxiety have established Minimal Clinically Important Differences [19], and this is therefore an important area for future research. 

This paper describes a service evaluation conducted in one general dental practice. The study found a reduction in child dental anxiety and established the feasibility of using these resources in a general dental practice setting for further research, preferably a randomised controlled trial. To establish the effectiveness of the guided self-help CBT resources, this randomised controlled trial should compare the intervention (delivered to dentally anxious children by general dental practitioners) with usual care, investigating the effect of the intervention on dental anxiety, Oral Health Related Quality of Life (OHRQoL), and use of pharmacological approaches. Ideally, the trial should assess both the clinical and cost-effectiveness of the intervention as well as include a process evaluation.

## 5. Conclusions

This is the first evaluation of the use of a guided self-help CBT resource in general dental practice. The resource reduced child dental anxiety and is of benefit to general dental practitioners in the dental treatment of this group of patients, who can be challenging to manage in general dental practice. As a service evaluation, this project lacked a control group so further evaluation of this approach is needed, ideally using a randomised controlled trial design. 

## Figures and Tables

**Table 1:** Children’s Experiences of Dental Anxiety Measure (CEDAM) interval scores at baseline and following children’s use of the guided self-help cognitive behavioural therapy (CBT) resource and dental treatment.

Number of Children	Mean Score (SD)	Mode Score	Minimum Score	Maximum Score
Baseline (N = 84)	20.3 (SD = 2.2)	18.5	16.0	25.8
Follow-up * (N = 84)	16.4 (SD = 2.2)	14.0	14.0	21.4

* On completion of dental treatment and following the use of the guided self-help CBT resources.

## Supplementary Materials

The following are available online at https://www.mdpi.com/2304-6767/7/2/36/s1, Figure S1: Message to dentist proforma.
