# Supplementary material for: Evaluation of Self-Help Cognitive Behavioural Therapy for Children’s Dental Anxiety in General Dental Practice
Source: Dent J (Basel). 2019 Apr 1;7(2):36. doi: 10.3390/dj7020036 (PMC6631013; doi:10.3390/dj7020036)
Supplement: Supplementary file 1 [file dentistry-07-00036-s001.pdf]

YOUR TEETH YOU ARE IN CONTROL

Message to Dentist

1) This is how worried I feel on a scale of 1 to 10 where 1 is not being scared at all. [ ]

2) These are the things I am worried about

----

3) This is how painful I think it might be on a scale of 1 to 10 where 1 is not being painful at all [ ]

4) This is what I'd like to happen

----

5) This is what I don't want to happen

----

6) Things I plan to do:

- ❑ Play music or an audiobook
- ❑ Imagine somewhere great
- ❑ Squeeze a stress ball
- ❑ Play a mind game in my head
- ❑ Breathe and relax
- ❑ Do some maths in my head

7) Things I want you to do:

- ❑ Talk to me or explain or show me what you are doing
- ❑ Tell me how long it will actually take
- ❑ Other --

8) Agree clear stop signals

If you want your dentist to stop, you need a way to let them know. This might be something like lifting up your left hand when you feel like a rest or you need to swallow. If you agree this with the dentist before you start treatment, it makes things much easier if you need a break.

Sometimes if your dentist is at a key point it may be important they finish off before stopping. If so, you need to agree that they give an idea of how much longer is needed. That's the time to use one of your Tools.

MY STOP SIGNAL IS: --

Agreed by Me (sign) ------------------------------

Signed by (my dentist) ------------------------------

Date ------------------------------

Date ------------------------------

Don't forget to take this message with you to your dentist next time you go. You can write a new message each time you visit if you want.

This guide presents independent research funded by the National Institute for Health Research (NIHR) under its Research for Patient Benefit (RfPB) Programme (Grant Reference Number PB-PG-1111-26029). The views expressed are those of the authors and not necessarily those of the NHS, the NIHR or the Department of Health.

Produced by Five Areas Limited for Sheffield Teaching Hospitals NHS Foundation Trust. www.fiveareas.com
Copyright (2015). All rights reserved. Content used under licence from Five Areas Ltd.

YOUR TEETH YOU ARE IN CONTROL

How did it really go?

When your treatment is nearly over, here are some questions that will help you give honest feedback. Your dentist will want that – so say it as it is. Think back on your experience and how things went. There may be things to ask your dentist to do differently next time - and now's the time to ask.

Here are some phrases you could use next time you talk to your dentist.

When you did this, it made me feel:

The best things that worked today were:

How painful actually was it on a scale from 1 to 10 where 1 is not being painful at all? [ ]

Feedback from your dentist. Ask your dentist to write how well they thought you did here:

Now, write down anything you don't want to happen again

How worried might I be next time on a scale from 1 to 10 where 1 is not being at all worried? [ ]

So, what have you learnt?

Tick the things that work for you…

- ❑ Having a plan.
- ❑ Writing a Message to Dentist
- ❑ Using a stop signal.
- ❑ Having something to look forward to afterwards.
- ❑ Using my Tools.
- ❑ Taking things step by step.
- ❑ Asking for clear information.

Other: (write in) ____________________

You've done well

It's good to recognise that with a small reward. It's your choice – but be realistic. We're not talking a holiday abroad.

A treat might be to:

- Play your favourite game for an extra hour that evening.
- Watch your favourite TV programme.
- Stay up a little later tonight.
- Choose your next meal.
- Download a new app.
- Buy some music.

Not sweets or fizzy drinks ☺
Choose something that will make you feel good. You deserve it.

My reward will be: ____________________
